# Finding Meanings in Low Dimensional Structures: Stochastic Neighbor Embedding Applied to the Analysis of *Indri indri* Vocal Repertoire

**DOI:** 10.3390/ani9050243

**Published:** 2019-05-15

**Authors:** Daria Valente, Chiara De Gregorio, Valeria Torti, Longondraza Miaretsoa, Olivier Friard, Rose Marie Randrianarison, Cristina Giacoma, Marco Gamba

**Affiliations:** 1 Dipartimento di Scienze della Vita e Biologia dei Sistemi, Università degli Studi di Torino, 10123 Torino, Italy; chiara.degregorio@unito.it (C.D.G.); valeria.torti@unito.it (V.T.); longondraza.miaretsoa@unito.it (L.M.); olivier.friard@unito.it (O.F.); cristina.giacoma@unito.it (C.G.); marco.gamba@unito.it (M.G.); 2 Group d’Etude et de Recherche sur les Primates de Madagascar, Antananarivo 101, Madagascar; sissienarda@yahoo.fr; 3 Mention d’Anthropobiologie et de Développement Durable (MADD), Université d’Antananarivo, Antananarivo 101, Madagascar

**Keywords:** lemurs, vocal communication, unsupervised analyses

## Abstract

**Simple Summary:**

The description of the vocal repertoire represents a critical step before deepening other aspects of animal behaviour. Repertoires may contain both discrete vocalizations—acoustically distinct and distinguishable from each other—or graded ones, with a less rigid acoustic structure. The gradation level is one of the causes that make repertoires challenging to be objectively quantified. Indeed, the higher the level of gradation in a system, the higher the complexity in grouping its components. A large sample of *Indri indri* calls was divided into ten putative categories from the acoustic similarity among them. We extracted frequency and duration parameters and then performed two different analyses that were able to group the calls accordingly to the a priori categories, indicating the presence of ten robust vocal classes. The analyses also showed a neat grouping of discrete vocalizations and a weaker classification of graded ones.

**Abstract:**

Although there is a growing number of researches focusing on acoustic communication, the lack of shared analytic approaches leads to inconsistency among studies. Here, we introduced a computational method used to examine 3360 calls recorded from wild indris (*Indri indri*) from 2005–2018. We split each sound into ten portions of equal length and, from each portion we extracted spectral coefficients, considering frequency values up to 15,000 Hz. We submitted the set of acoustic features first to a t-distributed stochastic neighbor embedding algorithm, then to a hard-clustering procedure using a k-means algorithm. The t-distributed stochastic neighbor embedding (t-SNE) mapping indicated the presence of eight different groups, consistent with the acoustic structure of the a priori identification of calls, while the cluster analysis revealed that an overlay between distinct call types might exist. Our results indicated that the t-distributed stochastic neighbor embedding (t-SNE), successfully been employed in several studies, showed a good performance also in the analysis of indris’ repertoire and may open new perspectives towards the achievement of shared methodical techniques for the comparison of animal vocal repertoires.

## 1. Introduction

Recent technological innovations in many areas of animal behavioral research, allow the collection of huge, complex, and often high-dimensional data sets. These can be daunting to be analyzed and may fail to satisfy the assumptions required by common statistical models [1]. Still, despite the high-dimensionality, because of the redundancy and multicollinearity of variables, data can be reduced and represented by fewer features [1]. The data reduction, indeed, allows the decrease of the storage amount and that of computational time, an easier understanding of data distribution, the improvement of visualization, classification and clusterization of high dimensional data [1,2]. Moreover, the dropping of uninformative attributes may help to highlight the best predictors and to improve the model’s accuracy [1,3]. Dimensionality reduction can be performed with different kinds of procedures [1,2]: Classical methods like the metric multi-dimensional scaling (MDS) [4] and the principal components analysis [5] are fast and efficient but they may fail to identify the real structure of datasets when they contain a nonlinear configuration [6]. Both techniques also embed a cost function more reliable with the modeling of large dissimilarities rather than the small ones. Therefore, they may not provide a good visualization of data [6,7]. More recent methods, such as the stochastic neighbor embedding (SNE) [8] or the local linear embedding (LLE) [9], aim to represent the similarity structure of objects by involving a two-dimensional visualization, where the higher the similarity between pairs, the less the distance between them [7]. The SNE foundation is the modeling of pairwise similarities by transforming Euclidean distances into likelihoods of selecting neighbors [2] and, being centered on a probabilistic model, it uses different bi-dimensional spaces and combines them into a single model of similarity, therefore leading to a good visualization of data [7]. Still, albeit the latter, massive use of the SNE is prevented because of its “crowding problem” (the tendency to pack points together in the center of the plan) and because it uses a cost function difficult to be optimized [10]. We used a variation of stochastic neighbor embedding [8] the t-distributed stochastic neighbor embedding (t-SNE) [10] that differs from the first one by using a symmetrized variant of the SNE cost function with simpler gradients as introduced by Cook, J.A. et al [7]. It also uses a Student’s t-distribution to compute the pairwise dissimilarities in low-dimensional space, instead of a Gaussian distribution [10]. The t-SNE heavy-tailed distribution allows confining both the optimization and the crowding problem of SNE, producing notably improved visualization [10]. Since its introduction, due to its flexibility, efficiency, and accuracy, various studies successfully applied the t-SNE and its extensions to the visualization and the classification of different kinds of objects: Paintings [11], single nucleotide polymorphisms (SNPs) [12], data collected by computer-aided diagnosis systems (CADx) [13], and high-dimensional cytometry data in mouse tumors [14]. t-SNE has also been employed in several studies investigating a wide range of acoustic aspects: To solve problems in the estimation and characterization of pitch content in musical audio [15], to examine similarities among words and phrases in natural language processing [16], to visualize relevant selected features of audio data [17], to characterize singing styles and to discriminate vocal and non-vocal contours [18], and to perform a dimensionality reduction in the building of an efficient technique of speaker recognition [19]. Still, this promising technique has hitherto rarely been applied to the study of animal behavior in general (stereotyped behavior of freely moving fruit flies, *Drosophila melanogaster*) [20], and never to investigate animals’ vocal behavior. However, vocal repertoires may represent an ideal model for this kind of analysis. Indeed, the sounds investigation often implies the analyses of huge, high-dimensional datasets [21]. We used t-SNE to analyze the vocal repertoire of *Indri indri*, the largest living lemur and the only one producing coordinated vocal displays. Nonetheless, the particular song is not the only noteworthy trait of the species, which also possesses an interesting vocal repertoire. Non-human primates’ vocal repertoires have been usually classified either as discrete (e.g., *Macaca fuscata* [22]; *Macaca Sylvanus* [23], with acoustically distinct call types clearly distinguishable from each other, or graded (e.g., *Cercopithecus diana* [24]; *Cercopithecus nictitans* [25]), when the acoustic structure of the vocalizations does not show neat boundaries between call types [26,27,28]. Yet, the dividing line between these two categories is not always clear and the classification of a whole repertoire as either continuous or discontinuous, may constitute an oversimplification [27,29], as repertoires may show both graded and discrete features (e.g., *Papio ursinus* [29]; *Cercopithecus neglectus*, *Cercopithecus campbelli, Cercocebus torquatus*, [30]), and the differentiation within vocal types may occur to varying degrees [31,32]. Traditionally, a large number of studies relied on the comparison of sounds similarity using clustering methods [33] based on acoustic features extracted from spectrograms. Still, although these algorithms showed good results in the classification of sounds, they could fail to describe the graded transition of call types that may occur in vocal repertoires [29]. Moreover, the gradation level is precisely one of the main reasons for the lack of consistency in vocal repertoire sizes assessments. Indeed, the higher the level of gradation, the higher the potential for information diffusion but also the higher the complexity in grouping the components of a system [28]. We expected to find a repertoire containing both graded and conspicuous signals [29,30] and, according to the call social function hypothesis, an acoustic variation of calls associated with their function [27,28,30,34]. Calls related to social contexts show the highest variation level when associated with affiliative value, while the highest level of stereotypy is associated with agonistic contexts (*Cercopithecus campbelli* [35]); alarm calls show an intermediate gradation level. Hence, we expected to find great flexibility in those calls having an affiliative social function, a rigid structure of signals associated with negative contexts, and an intermediate variation in the alarm calls. Accordingly, in agreement with Peckre and colleagues [28], we expected to find a clearer clusterization of discrete calls and a weaker grouping accuracy of graded ones. Finally, in agreement with the “social complexity–vocal complexity hypothesis” [30] and the social complexity hypothesis for communicative complexity [28], we expected indris to possess a small repertoire size if compared to that of other lemurs [21] or other primates [36] living in larger social groups.

## 2. Materials and Methods

### 2.1. Data Collection

We recorded spontaneous vocalizations of 18 groups of indris at four different forest sites: Six groups (1R, 2R, 3R, 5R, 6R, and XR) were recorded in Analamazaotra Special Reserve (18°56′ S, 48°25′ E), one group (1M) in Mantadia National Park (18°28′ S, 48°28′ E), three groups (ASF, YSF, and WSF) in Mitsinjo Forest Station (18°56′ S, 48°24′ E), eight groups (1MZ, 2MZ, 3MZ, 4MZ, 5MZ, 6MZ, 8MZ, and 10MZ) in Maromizaha Forest New Protected Area (18°56′ S, 48°27′ E). Data from all forest sites, apart from Maromizaha, were collected from 2005–2008. Indris inhabiting the Maromizaha forest were sampled from 2008–2018. Recordings were collected using a Sennheiser shotgun ME 66 and ME 67 (Sennheiser electronic GmbH & Co. KG, Wedemark, Hanover, Germany) and AKG CK 98 microphones (AKG Acoustics, Harman International Industries, Vienna, Austria). The signals were recorded at a sampling frequency rate of 44.1 kHz using a solid-state digital audio recorder: Marantz PMD671 (Marantz, Kew Gardens, NY, USA), SoundDevices 702 (Sound Devices, LLC, Reedsburg, WI, USA), Olympus S100 (Olympus Corporation, Shinjuku, Tokyo, Japan), or Tascam DR-100MKII 24 bit/96 kHz (TEAC Corporation, Montebello, CA, USA), with a 16-bit amplitude resolution. Vocalizations were recorded at a distance from 2–10 m since all the study groups were habituated, and all efforts were made to ensure that the microphone was oriented toward the vocalizing animal. Focal animal sampling [37] and the presence of individual-specific natural marks, allowed the attribution of each vocalization to a signaler. Only spontaneous utterances were recorded, avoiding the use of playback stimuli.

### 2.2. Acoustical Analysis

We visually inspected all recordings using spectrograms (Praat 6.0.28) (Phonetic Sciences, University of Amsterdam, Amsterdam, The Netherlands) [38] and then cut high-quality vocal emissions, normalized, saved into single files (*n* = 3360), and assigned to nine putative categories on the basis of their acoustic and spectrographic evaluation, according to the vocal types identified in a previous study [39]: Clacsons (*n* = 622), grunts (*n* = 1145), hums (*n* = 418), kisses (*n* = 296), long tonal calls (*n* = 31), roars (*n* = 62), short tonal calls (*n* = 44), wheezes (*n* = 150), and wheezing grunts (*n* = 297). Moreover, all indris within a familiar group participate in a chorusing song, mainly consisting of harmonic frequency modulated notes [40]. We also isolated units from the songs and grouped them in a tenth category (songbits, *n* = 295). Eight vocal types and 1275 vocalizations out of 3360 were included in a previous analysis [39]; wheezing grunts were previously identified [41] but not detected by Maretti and colleagues [39], and song units were not considered in that former repertoire description. For each call, we extracted spectral coefficients using a custom-made script in Praat [38]. The script first calculated the overall duration of a sound and then split it into ten portions of equal length. For each portion, the frequency range between 50 Hz and 15,000 Hz was divided into sets of frequencies called bins or bands (e.g., 50–500 Hz, 501–1000 Hz, 1001–1500 Hz, and 2001–2500 Hz). For each bin, we extracted the energy value using the function ‘Get band energy’ in Praat. The resulting dataset contained 3360 samples with 151 attributes for each; one hundred and fifty parameters were frequency parameters, the last was the duration of sounds.

### 2.3. Acoustic Embedding and Classification Procedure

We embedded the spectral features vectors into a bi-dimensional space using t-distributed stochastic neighbor embedding [10] with a Barnes-Hut implementation, using the Rtsne package [42] in R (R Core Team 2018; version 3.5.1, R Foundation for Statistical Computing, Vienna, Austria) [43]. We then used the t-SNE model (perplexity = 40, theta = 0.5, dims = 2) to group the cases, using k-means clustering [44]. t-SNE was also used for data visualization. We then used the WEKA 3.8 (Waikato Environment for Knowledge Analysis) [45] machine learning tool for the implementation of two classification algorithms. We applied multi-layer perceptron (MLP) [46,47], for the quantitative categorization of both the cluster assignment and the vocal type prediction, using the 67% of the dataset to train the neural network. We then computed two mean confusion matrices, one from the vocal types assigned a priori and the classes predicted by the MLP, the other one from the cluster assigned with the t-SNE procedure and the classes predicted by the network. Finally, to compare the results of the t-SNE cluster assignment to that of a k-means clustering (with *k* = 7, calculated through an average silhouette width) performed on a dataset reduced with a principal components analysis (and indicating six principal components), we applied a third network for the quantitative categorization of the cluster assignment.

## 3. Results

### 3.1. t-SNE Mapping

**Figure 1:**
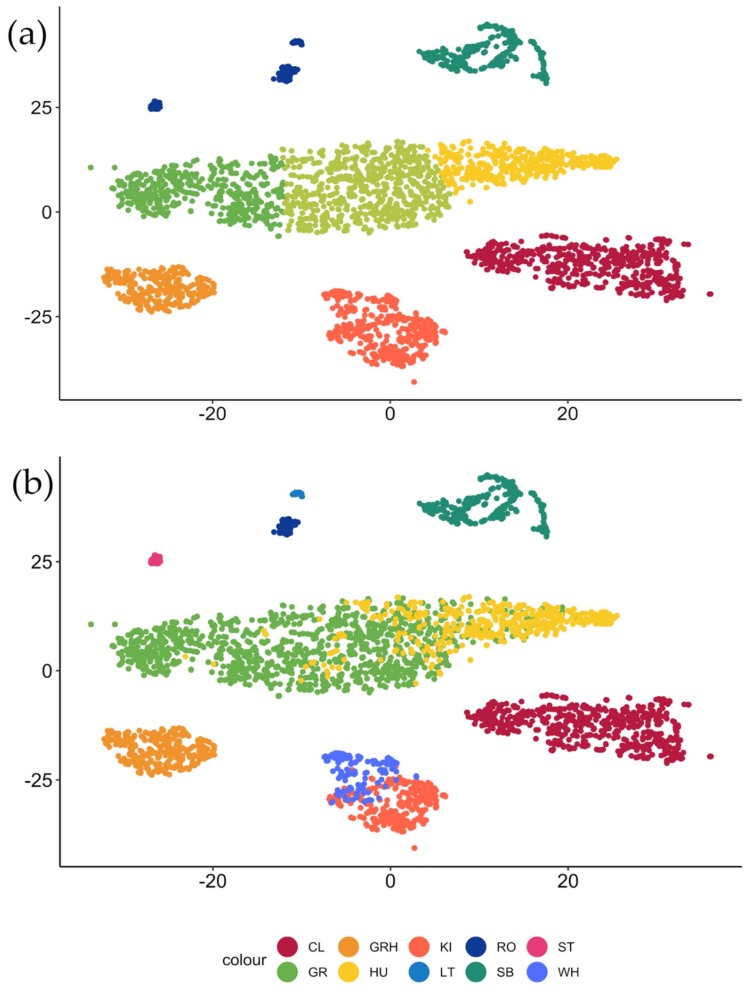
Bi-dimensional representation of the whole sample of sounds obtained initializing the t-distributed stochastic neighbor embedding (t-SNE) algorithm with perplexity = 40 and theta = 0.5. (**a**) Output of the t-SNE mapping combined with the k-means clustering results. (**b**) Remapping of the t-SNE output with the a priori classification and distribution of the vocal types in the clouds identified by the algorithm (cl = clacsons, gr = grunts, grh = wheezing grunts, hu = hums, ki = kisses, lt = long tonal calls, ro = roars, sb = songbits, st = short tonal calls, and wh = wheezes).

**Figure 2:**
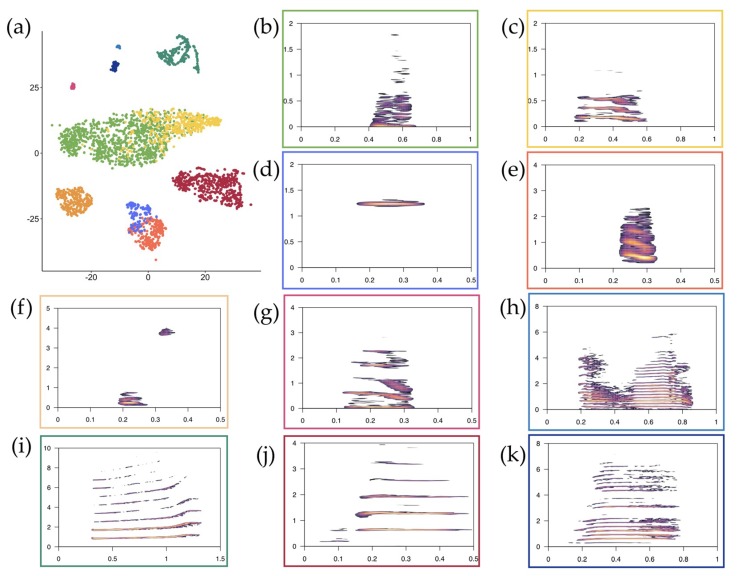
(**a**) Distribution of the vocal types in the clouds identified by the t-SNE map and their spectrographic representation: (**b**) Grunt, (**c**) hum, (**d**) wheeze, (**e**) kiss, (**f**) wheezing grunt, (**g**) short tonal call, (**h**) long tonal call, (**i**) songbit, (**j**) clacson, and (**k**) roar. Almost all classes (except kisses and wheezes and hums and grunts) were well separated. Spectrograms—frequency (kHz) on the y-axis and time (s) on the x-axis—were obtained with a Hanning window, 512 samples, 0% overlap, and no zero-padding using the Seewave package [48,49].

The t-SNE algorithm identified eight clouds (Figure 1a), we, therefore, performed a k-means clustering with *k* = 8. As highlighted in Figure 1a,b, the analysis recognized eight different clusters; all groups but three were consistent with the acoustic structure of the a priori identification. Cluster one, two, and three exclusively contain a vocal type each: Wheezing grunts (Figure 2f, Figure S1e), songbits (Figure 2i, Figure S2c), and clacsons (Figure 2j, Figure S2b), respectively (Table 1). Kisses and wheezes (Figure 2d,e, Figure S1c,d) were grouped in cluster five (66.37% and 33.63%, respectively), while grunts and hums (Figure 2b,c, Figure S1a,b) were both included in clusters four, seven, and eight. Specifically, cluster four contained mainly grunts (85.04%) and a small percentage of hums (14.96%); cluster seven, just as cluster four, comprised mostly grunts (99.00%). Conversely, cluster eight included a great portion of hums (82.06%) and a smaller part of grunts (17.94%). Short tonal (Figure 2g, Figure S1f), long tonal calls (Figure 2h, Figure S2d), and roars (Figure 2k, Figure S2a), although emerging as single clouds in the map, were grouped together in cluster six (respectively, 22.63%, 45.36%, and 32.12%, Table 1).

### 3.2. Call Recognition

For the quantitative categorization of both the cluster assignment and the vocal type prediction, the network we selected, trained for 500 iterations yielded the best performance by using a learning rate = 0.2 and momentum = 0.2. The correct attribution for the vocal type prediction achieved the 85.57% (*n* = 949, kappa statistic: 0.820; mean absolute error: 0.034; root mean squared error: 0.157; Table 2). The network recognized all vocal categories with percentages of correct classification ranging from 58.76% for the wheezing grunts to 100.00% for the long tonal calls and roars. Clacsons and songbits were almost totally correctly classified (99.03% and 98%, respectively). The classification of grunts achieved lower performances (84.25%), hums (84.56%), kisses (77.89%), short tonal calls (75.00%), and wheezes (78.57%, Table 3).

The model built for the cluster assignment showed better results. A total of 1109 instances were correctly classified in 1059 cases (95.49%, kappa statistic: 0.947; mean absolute error: 0.016; root mean squared error: 0.088; Table 4). The network recognized all clusters with high percentages of correct classification (Table 5). Five groups (clusters 1, 3, 5, and 6) were entirely correctly classified, with a rate of correct assignment of 100%. The last three groups’ classification showed almost as good results. The lowest performance was achieved by cluster 4 that was correctly classified in 85.35% of cases. Cluster 7 and cluster 8 showed the highest results: The first was correctly classified in 96.92%, while the second reached 95% of correct assignation. These groups, containing almost the totality of cases misclassified with respect to the clustering assignment, corresponded to the clusters showing a less homogeneous composition (Table 1): Cluster 4 and 7, contained mainly grunts (85.04% and 99.00%, respectively) and smaller percentages of hums (14.96% and 1%, respectively). On the other side, cluster 8 included a great portion of hums (82.06%) and a smaller part of grunts (17.94%). The third model, built using the PCA-based clustering as class, showed slightly weaker results when compared to the t-SNE model (93.05% vs. 95.49%; kappa statistic: 0.897; mean absolute error: 0.02; root mean squared error: 0.13).

## 4. Discussion

We described the use of a computationally simple but powerful method applied in the automatic recognition of acoustic signals. The t-SNE embedding and the use of MLP allowed an efficient analytical performance: Our results indicate that it was possible to automatically identify vocal types by using a dataset consisting of high-dimensional vector representations of objects, assigning similarities between those objects as conditional probabilities [10]. Still, although both t-SNE [15,16,17,18,19] and neural networks [50,51] are widely used to analyze acoustic characteristics in a wide range of research fields, ours represents the first attempt to combine these kinds of computational tools and apply them to the identification of vocal repertoire in nonhuman primates. Our findings support what was found in a previous analysis on indris’ vocal repertoire [39]. Indeed, our analysis confirmed the presence of the eight call types emerged in the study, but we also identified two further categories: The songbits, consisting of all units given by an indri during the choral song of the group, were not considered to the purposes of the qualitative assessment of *Indri indri* vocal repertoire; and the wheezing grunts [41], particular vocalizations given after agonistic physical interactions (pers. obs.), were not detected by Maretti and colleagues [39]. Albeit our analysis allowed us to easily distinguish the different vocal types, the algorithm’s map contained some points clustered within the wrong class. Most of these points correspond to sounds belonging to vocal classes showing a certain degree of gradation one another and therefore may be difficult to be identified [29]. In particular, we found an overlay between hums and grunts and kisses and wheezes. Hums (also known as weak grunts) [52] and grunts are both low-frequency and low-intensity calls; hums show a more defined harmonic structure when compared to grunts that, in contrast, show a clearer and low-pitched pulsed structure [39].

Furthermore, hums serve as group-cohesion calls [39] and their gradation level is following what was found in Campbell’s monkeys (*Cercopithecus campbelli*), where calls associated with high affiliative social values show an elevated gradation level [35]. The great gradation in these calls may allow for flexible usage and the encoding of multiple elements of information, in agreement with the findings of Keenan and colleagues on *Cercopithecus campbelli* [27]. Overall, our results are in line with findings on red-capped mangabeys (*Cercocebus torquatus*), whose contact calls show more acoustic dissimilarity than long-distance and alarm signals [53], in contrast with findings on chacma (*Papio ursinus*), olive (*P. anubis*), and Guinea (*P. papio*) baboons, whose loud calls are more differentiated than grunts [54]. Kisses and wheezes, on the other hand, are both brief medium-intensity vocalizations, often uttered together (85% of cases) [39]. They are stress-related vocalizations that can be emitted as contact-rejection call, before a song, or in response to anxiety-causing stimuli [39,41,55]. In our analysis, the categories identification relied on a human visual assessment, and the vocal classes grouping, although supported by our findings, may imply dissimilarities perceived by humans but not necessarily by the species [56,57]. Moreover, in agreement with what was hypothesized, our results indicated the presence of signals showing features of both conspicuousness and gradedness, as found in other primate species [27,29,30] and the analysis showed a stronger accuracy in the classification of discrete calls, than that of graded ones [28]. We expected the variation of calls to be associated with their social function [35], with calls having affiliative value showing the highest variation level, calls associated with agonistic contexts showing the highest stereotypy, and alarm calls showing an intermediate gradedness. This prediction was not entirely supported by our results, as we found the two alarm calls (roars and clacsons), well separated from one another. The result seems instead to be in line with studies on calls referentiality [58,59,60]. Additionally, the roars were grouped together with long tonal and short tonal calls; these three vocal types are the only with a chaotic component [39] and the result may depend by their spectral features, known to affect the vocalization recognition [21,61].

Finally, in agreement with the social complexity–vocal complexity hypothesis [30] and the social complexity hypothesis for communicative complexity [28], the vocal repertoire size is directly proportional to the group size. We expected indris to possess a small repertoire size compared to that of other lemurs [21] and other primates [36] living in larger social groups. A ten-categories vocal repertoire and an average group size of four to six individuals, seemed not to be in line with this theory, in accordance with findings on *Eulemur rubriventer*, owning a vocal repertoire of 14 vocal types and a group size of about three individuals [21]. Notably, both species also show a stable social monogamous organization [62,63], in agreement with the hypothesis stating that the diversity in communication signals may be favored by an egalitarian social structure or a stable social group [64]. These findings are also in agreement with the studies on Asian colobines *Pygathrix nemaeus* [65] and *Nasalis larvatus* [66,67], showing a repertoire size smaller or similar to that of indris, compared to an average group size sometimes even significantly higher.

## 5. Conclusions

As earlier hypothesized, the vocal repertoire structure may be determined by both the species’ environment and social structure [68]. This could also be for the indris’ case, where the presence of loud and discrete calls, like alarm calls [27,68] and even the song, may have evolved to cope with a noisy environment and poor visual ranges, like that of dense rainforests, to reduce the misinterpretation of signals in the long-distance and even in inter-group communication. On the other side, contact calls and in general vocalizations that may serve the intra-group and short-range communication, do not have to face such kinds of obstacles and may show a more graded structure.

## Figures and Tables

**Table 1:** Distribution of the vocal types in the eight clusters (expressed in %). Cl: clacsons; GR: grunts; GRH: wheezing grunts; HU: hums; KI: kisses; LT: long tonal calls; RO: roars; SB: songbits; ST: short tonal calls; WH: wheezes.

Cluster	CL	GR	WG	HU	KI	LT	RO	SB	ST	WH
1st	0.00	0.00	100.00	0.00	0.00	0.00	0.00	0.00	0.00	0.00
2nd	0.00	0.00	0.00	0.00	0.00	0.00	0.00	100.00	0.00	0.00
3rd	100.00	0.00	0.00	0.00	0.00	0.00	0.00	0.00	0.00	0.00
4th	0.00	85.04	0.00	14.96	0.00	0.00	0.00	0.00	0.00	0.00
5th	0.00	0.00	0.00	0.00	66.37	0.00	0.00	0.00	0.00	33.63
6th	0.00	0.00	0.00	0.00	0.00	22.63	45.26	0.00	32.12	0.00
7th	0.00	99.00	0.00	1.00	0.00	0.00	0.00	0.00	0.00	0.00
8nd	0.00	17.94	0.00	82.06	0.00	0.00	0.00	0.00	0.00	0.00

**Table 2:** Vocal type assignment detailed accuracy by class. TP rate: Rate of true positives; FP rate: Rate of false positives; precision: Proportion of instances that are truly of a class divided by the total instances classified as that class; F-measure: Combined measure for precision and recall; ROC area: Receiver operating characteristics measurement area; PRC area: Precision recall area.

Vocal Type	TP Rate	FP Rate	Precision	Recall	F-Measure	MCC	ROC Area	PRC Area
CL	0.99	0.00	0.99	0.99	0.99	0.988	1.00	1.00
GR	0.82	0.08	0.84	0.82	0.83	0.74	0.94	0.88
GRH	0.71	0.04	0.59	0.71	0.64	0.61	0.96	0.65
HU	0.83	0.02	0.85	0.83	0.84	0.81	0.98	0.90
KI	0.79	0.02	0.78	0.79	0.78	0.76	0.98	0. 87
LT	1.00	0.00	1.00	1.00	1.00	1.00	1.00	1.00
RO	0.81	0.00	1.00	0.81	0.90	0.90	1.00	0.98
SB	1.00	0.02	0.98	1.00	0.99	0.99	1.00	1.00
ST	0.69	0.00	0.75	0.70	0.72	0.72	0.98	0.76
WH	0.75	0.01	0.79	0.75	0.77	0.76	0.95	0.84
Weighted Average	0.86	0.04	0.86	0.86	0.86	0.82	0.97	0.90

**Table 3:** Confusion Matrix on vocal type prediction. Cl: clacsons; GR: grunts; GRH: wheezing grunts; HU: hums; KI: kisses; LT: long tonal calls; RO: roars; SB: songbits; ST: short tonal calls; WH: wheezes.

Classified As	A	B	C	D	E	F	G	H	I	J
CL	99.03	0.00	0.00	0.00	0.00	0.00	0.00	0.00	0.00	4.76
GR	0.00	84.25	38.14	14.09	10.53	0.00	0.00	0.00	0.00	4.76
GRH	0.00	4. 99	58.76	0.00	4.21	0.00	0.00	0.00	0.00	0.00
HU	0.00	6.30	1.03	84.56	0.00	0.00	0.00	0.00	0.00	2.38
KI	0.00	3.15	1.03	0.67	77.89	0.00	0.00	0.00	16.67	9.52
LT	0.00	0.00	0.00	0.00	0.00	100.00	0.00	0.00	0.00	0.00
RO	0.97	0.00	0.00	0.00	0.00	0.00	100.00	2.00	0.00	0.00
SB	0.00	0.00	0.00	0.00	0.00	0.00	0.00	98.00	0.00	0.00
ST	0.00	0.79	0.00	0.67	0.00	0.00	0.00	0.00	75.00	0.00
WH	0.00	0.52	1.03	0.00	7.37	0.00	0.00	0.00	8.33	78.57

**Table 4:** Cluster assignment detailed accuracy by class. TP rate: Rate of true positives; FP rate: Rate of false positives; precision: Proportion of instances that are truly of a class divided by the total instances classified as that class; F-measure: Combined measure for precision and recall; ROC area: Receiver operating characteristics measurement area; PRC area: Precision recall area.

Cluster	TP Rate	FP Rate	Precision	Recall	F-Measure	MCC	ROC Area	PRC Area
3rd	1.00	0.00	1.00	1.00	1.00	1.00	1.00	1.00
1st	1.00	0.00	1.00	1.00	1.00	1.00	1.00	1.00
4th	0.96	0.05	0.85	0.96	0.90	0.88	0.99	0.97
7th	0.83	0.00	0.97	0.83	0.90	0.88	1.00	0.98
8th	0.88	0.01	0.95	0.88	0.92	0.91	1.00	0.98
5th	1.00	0.00	1.00	1.00	1.00	1.00	1.00	1.00
6th	1.00	0.00	1.00	1.00	1.00	1.00	1.00	1.00
2nd	1.00	0.00	1.00	1.00	1.00	1.00	1.00	1.00
Weighted Average	0.95	0.01	0.96	0.95	0.95	0.95	1.00	0.99

**Table 5:** Confusion Matrix on cluster assignment.

Classified as	A	B	C	D	E	F	G	H
3rd	100.00	0.00	0.00	0.00	0.00	0.00	0.00	0.00
1st	0.00	100.00	0.00	0.00	0.00	0.00	0.00	0.00
4th	0.00	0.00	85.35	3.08	5.00	0.00	0.00	0.00
7th	0.00	0.00	9.16	96.92	0.00	0.00	0.00	0.00
8th	0.00	0.00	5.49	0.00	95.00	0.00	0.00	0.00
5th	0.00	0.00	0.00	0.00	0.00	100.00	0.00	0.00
6th	0.00	0.00	0.00	0.00	0.00	0.00	100.00	0.00
2nd	0.00	0.00	0.00	0.00	0.00	0.00	0.00	100.00

## Supplementary Materials

The following are available online at https://www.mdpi.com/2076-2615/9/5/243/s1, Figure S1: Sound spectrograms of *Indri indri* vocal types (I), Figure S2: Sound spectrograms of *Indri indri* vocal types (II).
