# Supplementary material for: Finding Meanings in Low Dimensional Structures: Stochastic Neighbor Embedding Applied to the Analysis of Indri indri Vocal Repertoire
Source: Animals (Basel). 2019 May 15;9(5):243. doi: 10.3390/ani9050243 (PMC6562776; doi:10.3390/ani9050243)
Supplement: Supplementary file 1 [file animals-09-00243-s001.zip › SM_OK/FigureS1.pdf]

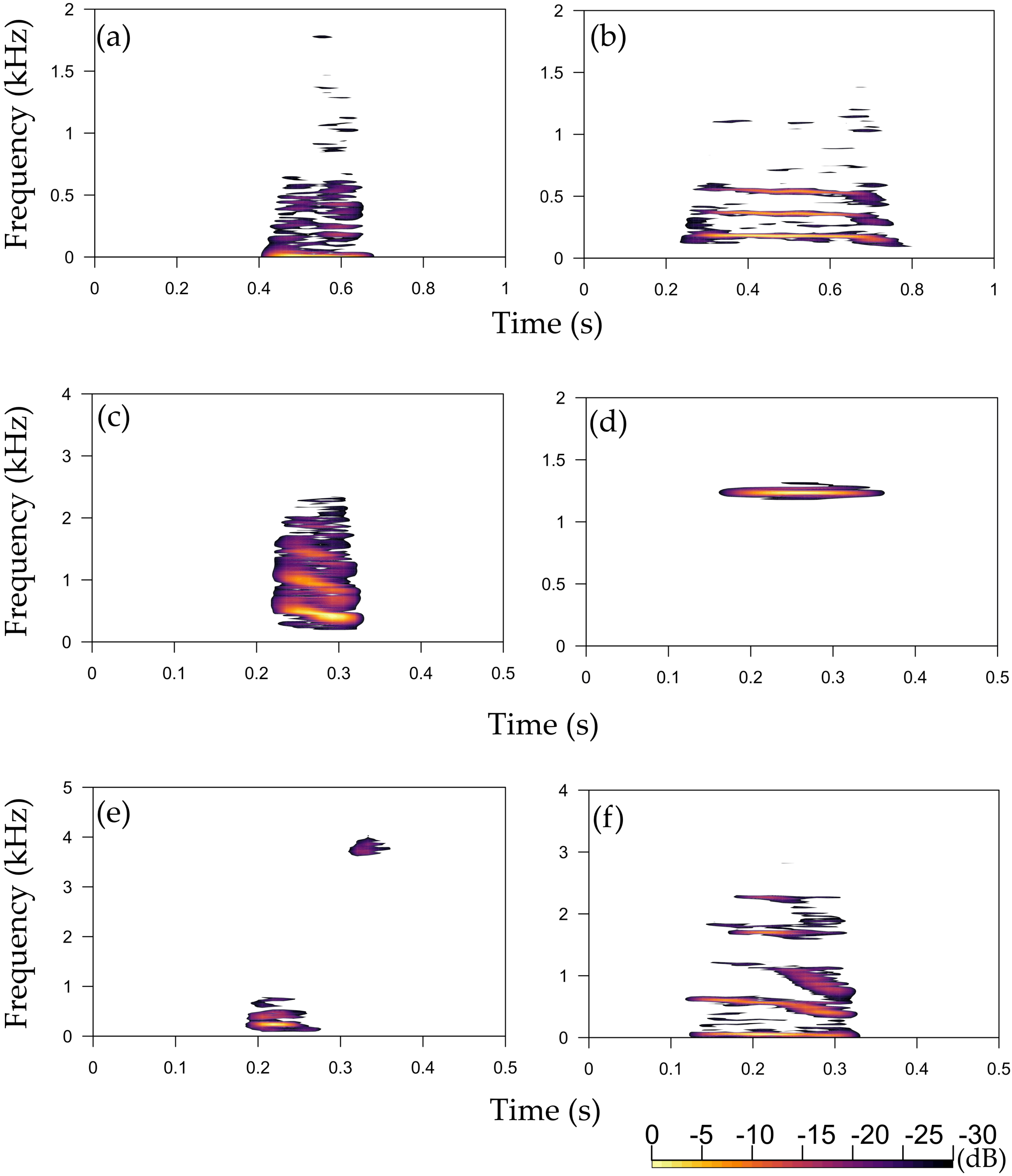

Figure S1: Sound spectrograms of *Indri indri* vocal types. **(a)** Grunt; **(b)** Hum; **(c)** Kiss; **(d)** Wheeze; **(e)** Wheezing grunt; **(f)** Short tonal call. All spectrograms were generated in R [43] through the Seewave package [48,49], using a Hanning window, 512 samples, 0% overlap, no zero-padding. Spectrograms of Grunt **(a)** and Hum **(b)** show a different time scale (1s) compared to the other (0.5s).
