# Supplementary figures and images for: Finding Meanings in Low Dimensional Structures: Stochastic Neighbor Embedding Applied to the Analysis of Indri indri Vocal Repertoire
Source: Animals (Basel). 2019 May 15;9(5):243. doi: 10.3390/ani9050243 (PMC6562776; doi:10.3390/ani9050243)

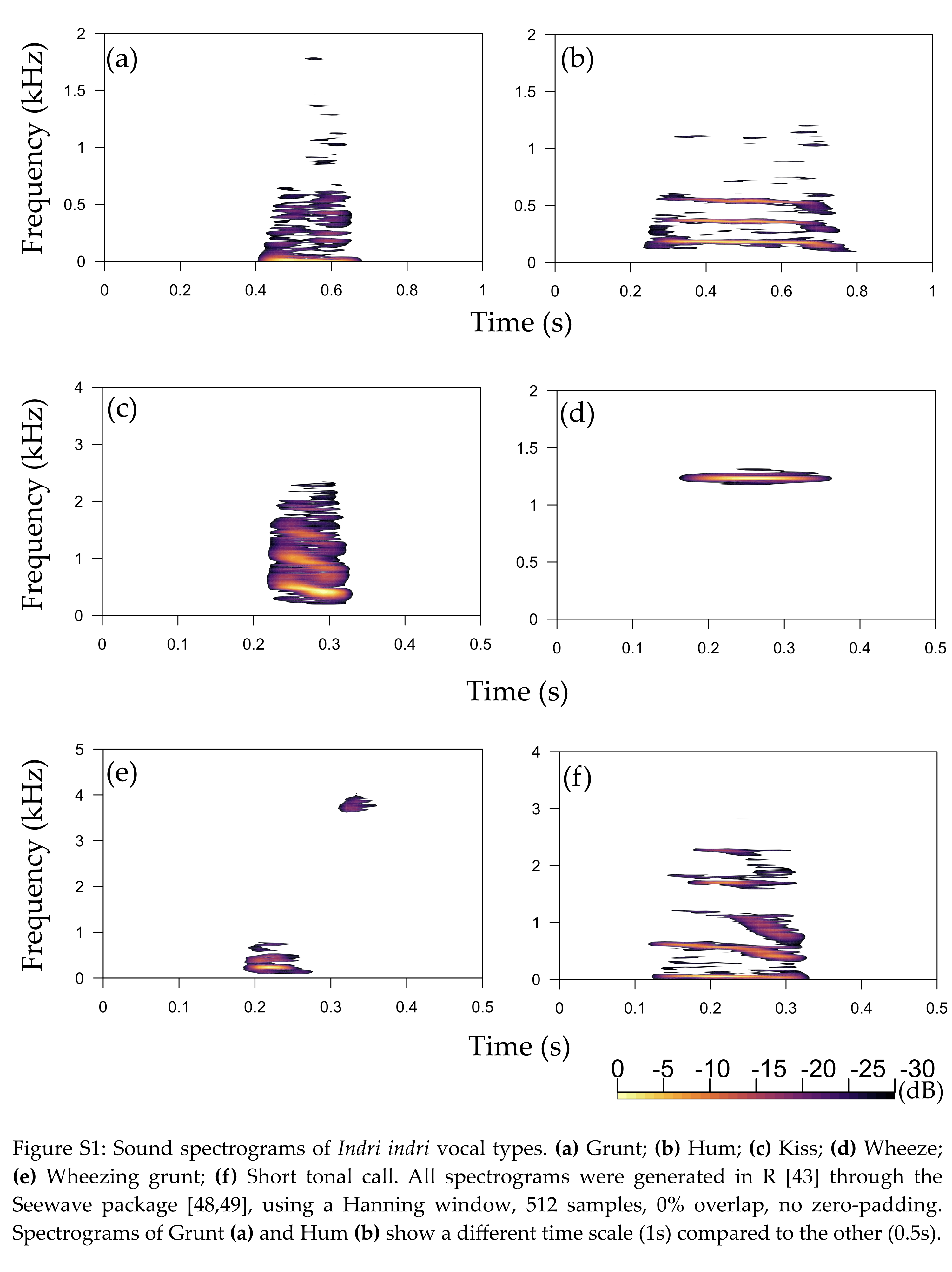

Supplement: Supplementary file 1 [file animals-09-00243-s001.zip › SM_OK/FigureS1.tiff]

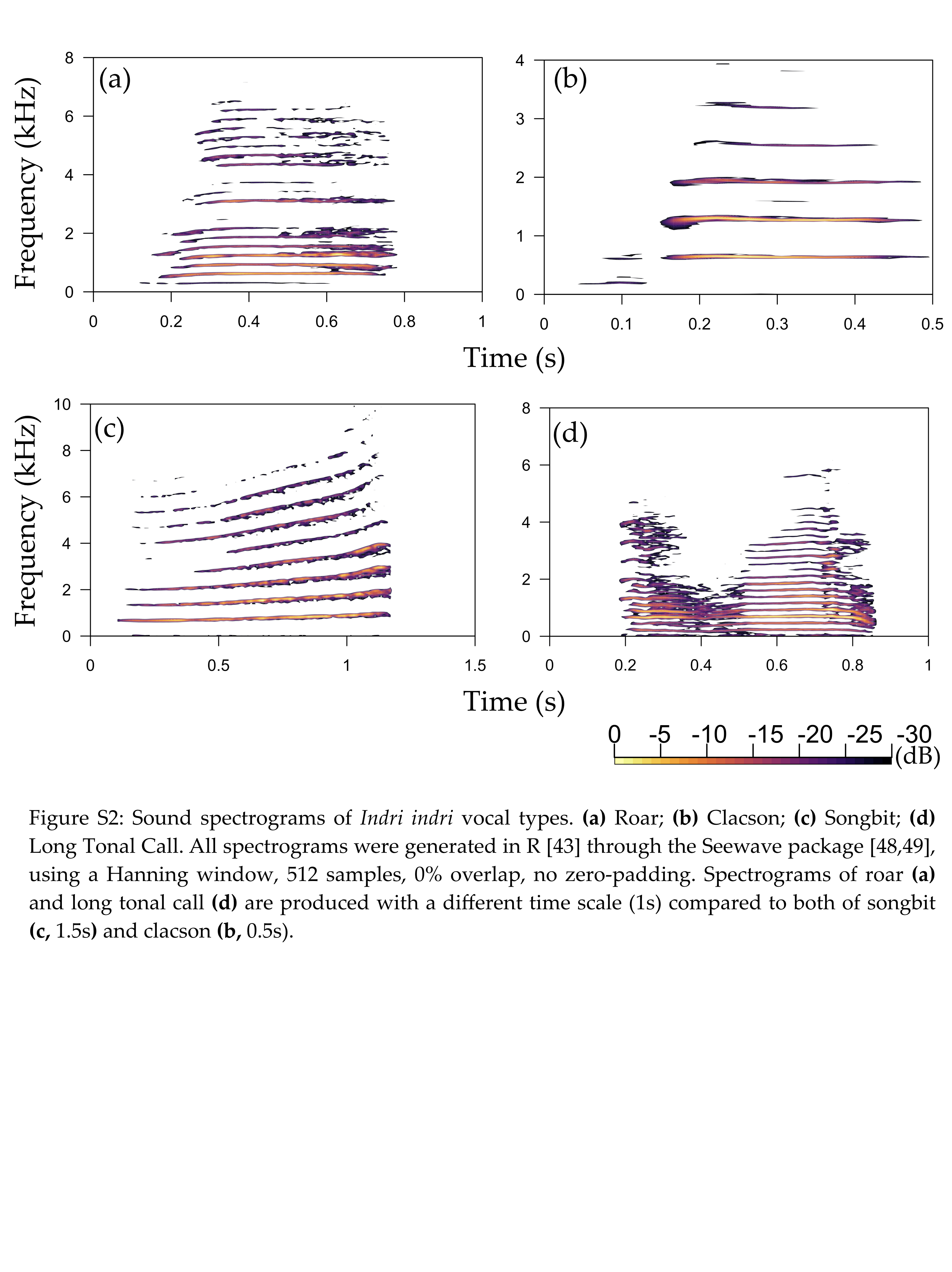

Supplement: Supplementary file 1 [file animals-09-00243-s001.zip › SM_OK/FigureS2.tiff]
